# Uropathogenic *Escherichia coli* (UPEC)-Associated Urinary Tract Infections: The Molecular Basis for Challenges to Effective Treatment

**DOI:** 10.3390/microorganisms11092169

**Published:** 2023-08-28

**Authors:** Shane Whelan, Brigid Lucey, Karen Finn

**Affiliations:** 1 Department of Biological Sciences, Munster Technological University, Bishopstown, T12 P928 Cork, Ireland; swhelan1@mycit.ie; 2 Department of Analytical, Biopharmaceutical and Medical Sciences, Atlantic Technological University Galway City, Dublin Road, H91 T8NW Galway, Ireland

**Keywords:** uropathogenic *E. coli*, biofilm formation, antibiotic resistance, mobile genetic elements

## Abstract

Urinary tract infections (UTIs) are among the most common bacterial infections, especially among women and older adults, leading to a significant global healthcare cost burden. Uropathogenic *Escherichia coli* (UPEC) are the most common cause and accounts for the majority of community-acquired UTIs. Infection by UPEC can cause discomfort, polyuria, and fever. More serious clinical consequences can result in urosepsis, kidney damage, and death. UPEC is a highly adaptive pathogen which presents significant treatment challenges rooted in a complex interplay of molecular factors that allow UPEC to evade host defences, persist within the urinary tract, and resist antibiotic therapy. This review discusses these factors, which include the key genes responsible for adhesion, toxin production, and iron acquisition. Additionally, it addresses antibiotic resistance mechanisms, including chromosomal gene mutations, antibiotic deactivating enzymes, drug efflux, and the role of mobile genetic elements in their dissemination. Furthermore, we provide a forward-looking analysis of emerging alternative therapies, such as phage therapy, nano-formulations, and interventions based on nanomaterials, as well as vaccines and strategies for immunomodulation. This review underscores the continued need for research into the molecular basis of pathogenesis and antimicrobial resistance in the treatment of UPEC, as well as the need for clinically guided treatment of UTIs, particularly in light of the rapid spread of multidrug resistance.

## 1. Introduction

Urinary tract infections are one of the most common bacterial infections, with an estimated 400 million cases and 230,000 deaths worldwide in 2019 [1]. Some 50% of women will experience a UTI at least once in their lifetime, with recurrence being common [2]. Furthermore, UTIs are one of the most common infections reported in older adults, being second only to respiratory infections in hospitalised patients over 65 [3]. The economic burden of UTIs is high, representing 6% of medical visits in the United States and costing approximately USD 1.6 billion annually [4]. A European study which sought to determine the cost associated with complicated urinary tract infection in eight countries with a high prevalence of multidrug-resistant Gram-negative bacteria found that the average cost of treatment was EUR 5700 per case [5]. An Irish study from 2014 estimated the primary care cost of UTI in Ireland to be EUR 19.2 million annually [6]. UTI infections fall into distinct clinical categories as defined by the European Association of Urology (EAU), which in turn guides treatment [7]. Uncomplicated UTIs are described as infections involving non-pregnant, pre-menopausal women with no known relevant urological abnormalities or comorbidities, while complicated UTIs are those affecting men, pregnant women, patients with anatomical or functional abnormalities, renal disease, indwelling urinary catheters and those with certain immunosuppressive co-morbidities [7]. A recurrent UTI (R-UTI) is defined as two or more uncomplicated or complicated UTI infections in six months, or three infections within one year that are new infections with separate organisms [8]. Certain conditions or abnormalities may predispose a person to recurrent infection. This can occur due to relapse and infection by the same persistent organism following unsuccessful treatment, but in the majority of cases it results from infection with a separate organism, post the resolution of symptoms [9].

The main cause of UTIs is infection by uropathogenic *Escherichia coli* (UPEC), responsible for 75% of uncomplicated UTIs and 65% of complicated UTIs [10]. UPEC form part of a group of pathogenic *E. coli* known as extraintestinal pathogenic *E. coli* (ExPEC), a collection of four pathotypes that are each classified by their respective site of isolation, and have adopted virulence mechanisms that allow them to proliferate and cause disease in host sites outside of the intestinal tract [11,12]. ExPEC are separable from diarrheagenic *E. coli* (DEC), a group of six *E. coli* pathotypes which cause intestinal infections, and each possess a different combination of virulence characteristics [13]. Infection begins when bacteria that reside in the gut enter the urethra and colonise epithelial cells and eventually migrate upwards to colonise the bladder. The initiation of the host’s innate immune response to infection represents a complex cascade of events. Central to this response are pattern recognition receptors (PRRs). Notably among this family are Toll-like receptors (TLRs), which recognise pathogen-associated molecular patterns (PAMPs) [14]. TLRs are expressed across a number of cell types, including immune cells such as NK cells, macrophages, and leukocytes, as well as non-immune cells such as bladder, ureter, and kidney urothelial cells [15,16]. The 10 TLRs (TRL1-10) which are found in humans are distinguishable by the ligands they recognise and the resulting signalling pathways they activate [17]. Critical to the host’s immune response during invasion by UPEC is TLR4, the sensing receptor for Gram-negative lipopolysaccharide (LPS) [18]. Upon detection of LPS, TLR-4 activates the intracellular signalling pathway NF-κB which regulates the expression of many proinflammatory cytokines and chemokines as well as leukocyte attracting chemoattractants [19,20]. Further inflammation is stimulated by TLR-5, which responds to the presence of bacteria flagellin [21]. While the locally attracted phagocytes are effective at clearing extracellular pathogens, TLR-4 additionally mediates the expulsion of intracellular pathogens through cAMP-dependent exocytosis from bladder epithelial cells [22].

Nevertheless, UPEC have acquired an array of virulence factors to circumvent host immune mechanisms and expedite infection and disease. These factors include proteins related to motility and adhesion which allow bacterial cells to attach to host receptors [23,24]. Some of these adhesive surface proteins bind preferentially to kidney cells which predispose some UPEC to pyelonephritis [25]. Adhesions allow bacteria to resist expulsion via urination. As well as this, adherence to the uroepithelium is the first step in the formation of intracellular bacterial communities (IBCs), where bacterial cells can evade expulsion by TLR-4, proliferate within the uroepithelium, and infect nearby cells, leading to persistence within the urinary tract [26,27,28]. UPEC which more readily form biofilms are also more persistent and difficult to treat due to the innate ability of the biofilm to shield cells from antibiotic therapy [29]. Initial attachment to host cells through surface adhesions is an important determinant of a particular isolate’s ability to form a biofilm, and several studies have highlighted the differences in fimbria gene distribution between strong and weak biofilm formers [30,31]. Additionally, virulence factors and antibiotic resistance genes are more readily exchanged among bacteria while in a biofilm [32]. While adhesions play a significant role as fitness factors for UPEC during urinary tract colonisation, similarly, iron-chelating agents such as siderophores are crucial. These secreted molecules enable UPEC to sequester iron from the urinary tract, a vital element for various bacterial cell processes that is scarce in this specific environmental niche [33]. Genes which encode toxins are considered important virulence factors not only because they induce damage and morphological changes in host cells, but because they also assist in the evasion of the host’s immune defences [34,35,36]. The *E. coli* genome is highly plastic, and virulence factors can be transferred between strains by horizontal gene transfer, on large pathogenicity islands (PAI’s) within extrachromosomal plasmids [37,38] The transfer of virulence factors between pathotypes can result in hybrid strains, which include those that, although isolated from urinary tract infections and possess virulence factors associated with UPEC, also possess genes which are definitive of DEC pathotypes [39,40,41]. For example, UPEC/DEC strains have been isolated from urinary tract infections which carry the *aggR* gene, while others which were found to carry the *eae* gene have been reported [40,41]. The *eae* gene mediates the attachment of enteropathogenic *Escherichia coli* (EPEC) to epithelial cells [42], while the *aggR* gene is a transcriptional regulator which controls the expression of multiple virulence factors associated with enteroaggregative *Escherichia coli* (EAEC) [43]. These hybrids may have the ability to cause disease at multiple sites of infection. Some studies have looked to determine the frequency by which hybrid UPEC are isolated from UTIs, with reported figures between 2% and 10.5% [40,41,44]. 

When studying the genetics of *E. coli*, they can be divided using multiplex PCR into 8 major phylogroups, those being A, B1, B2, C, D, E, F, and *Escherichia* cryptic clade I [45]. The distribution of these groups varies between locations, between clinical and commensal strains, animal or human carriage, and distribution of resistance genes. A meta-analysis of human *E. coli* isolates from 26 different countries found that phylogroup A was the most prominent, with a median result of 36%, followed by B2 at 20%, and B1 at 16.4%. [46]. Virulent extra-intestinal strains studied from samples taken from south India show that the groups B2 and D are most common [47]. Specifically for UPEC, a meta-analysis of samples from Iran revealed that the phylogroup B2 is most prominent at 32%, while D, B1 and A make up 26%, 18%, and 8%, respectively [48]. In addition to phylogenetic typing, antigen serotyping, specifically the O (lipopolysaccharide), H (flagellum), and K (capsule) antigens are also used frequently for characterisation [49]. The O antigen is particularly variable, and currently there are 186 recognized O serotypes of *E. coli* [49]. Among these, the majority of UPEC belong to serogroups O1, O2, O4, O6, O7, O8, O14, O15, O16, O18, O21, O22, O25, O75, and O83 [50,51,52,53,54]. Among these O25, O15, and O8 are more frequently detected, although specific distribution is subject to geographical location and patient population [52,53,54,55,56]. Apropos to this, numerous investigations have established associations linking virulence, antibiotic resistance, and distinct serogroups [52,53,54,55,56], underscoring the utility of this serotyping approach for epidemiological surveillance across diverse locales.

For the treatment of uncomplicated UTIs, the EAU recommends the use of fosfomycin trometamol, nitrofurantoin, and pivmecillinam, while cephalosporins are recommended as alternative therapy (Table 1). Trimethoprim/trimethoprim-sulfamethoxazole is recommended if local resistance rates are <20% and it is also recommended for the treatment of men, with fluoroquinolones suggested in accordance with local susceptibility testing. For complicated UTIs it is recommended to use amoxicillin plus an aminoglycoside or a second-generation cephalosporin plus an aminoglycoside. Intravenous third-generation cephalosporins are advised for the empirical treatment of symptomatic complicated UTIs (Table 1). It is also recommended that ciprofloxacin and other fluoroquinolones not be used in patients from urology departments or in cases where the patient has used fluroquinolones in the last six months [7]. It has been shown that laboratory guided treatment is preferable to empirical treatment, as national or regional guidelines may not accurately reflect actual resistance rates in specific geographical locations [57].

## 2. Virulence Factors 

The ability of UPEC to colonise the bladder and resist antibiotic therapy is attributed to several virulence factors, many of which are also related to biofilm formation [26,58,59]. These include adhesion factors such as Type 1 fimbriae, P fimbriae, curli fibres, S fimbriae, F1C fimbriae, Dr fimbriae, afimbrial adhesins, and PapC [60,61]. These adhesion factors enable UPEC to adhere to and invade host cells and tissues, evade the host immune response, and colonise the urinary tract [62,63]. In addition, the extracellular matrix of UPEC biofilms is composed mainly of amyloid curli fibres, which are encoded by the curli operon [64]. Curli fibres play a crucial role in UPEC biofilm formation and the extracellular matrix they compose provides protection from environmental stresses such as antibiotics, host defences, and nutrient deprivation [65]. Toxins such as α-hemolysin, cytotoxic necrotizing factor, and serine protease autotransporter can cause tissue damage, proteolytically cleave immune cell complexes, and trigger morphological changes in host cells which facilitate bacterial persistence [34,35,36].

**Figure 1:**
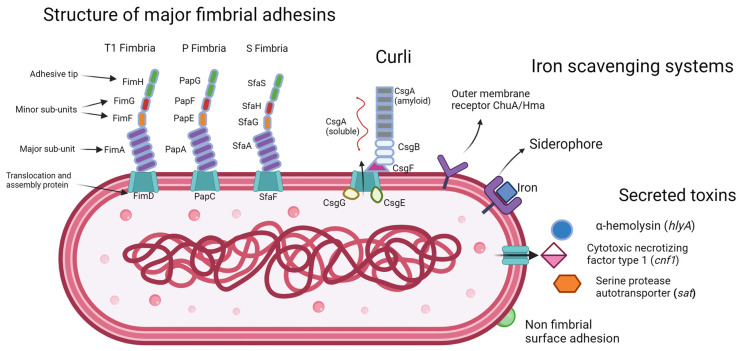
*E. coli* with a representative set of virulence factors of the UPEC pathotype.

Moreover, the ability of UPEC to acquire iron is highly dependent on siderophore-iron transporter proteins [33]. These proteins enable UPEC to scavenge iron from the host and other sources, which is essential for bacterial growth and survival in the environment of the urinary tract where iron levels are infinitesimal [33]. A representative set of the virulence factors that can be expressed by UPEC are shown in Figure 1.

Virulence factors such as adhesions and siderophores can coalesce into pathogenicity islands, be mobilised in transposons, and be transferred on plasmids [66]. This transfer of virulence factors enables UPEC to adapt and evolve rapidly in response to environmental pressures and facilitates the spread of antibiotic resistance genes among interspecies bacterial populations.

### 2.1. Adhesions

#### 2.1.1. Type 1 Fimbriae

Fimbria adhesions are extracellular protein structures that allow UPEC to attach to bladder cells and resist expulsion from the urinary system through the flow of urine. The best defined and most common fimbria adhesions are type 1fimbria (T1F). Genomic analysis of UPEC populations have reported prevalence of T1F between 86–100% [60,67,68]. Apart from initial attachment, T1F have been shown to mediate the invasion of bladder and uroepithelial cells, forming IBCs which can form persistent reservoirs of infection, are more resistant to antibiotics, and are speculated to contribute to recurrent infections [69,70]. 

T1F are encoded by the *fim* operon found in the majority of UPEC, but importantly, is not always expressed [71]. This operon contains nine genes, *fimA* to *fimI* with *fimA* expressing the main structural element, and *fimC* and *fimD* playing roles in the transport and assembly of the fimbriae [72]. *fimH* is responsible for facilitating binding of the fimbriae to host receptors, and has specificity for D-mannose-containing structures on host cells, often uroplakins [23]. The D-mannose-specific structures required for attachment are more numerous in the lower urinary tract than in the kidneys, so it was long believed that T1F do not play a role in pyelonephritis; however, more recent data suggest that T1F are still expressed in kidney infections, though they play the role of facilitating inter-bacterial adhesion and biofilm formation, rather than attachment and host cell invasion [73].

Expression of T1F is controlled by a reversible process called phase variation; a process by which the expression of bacterial surface factors, primarily adhesions, can be switched on or off [74]. Phase variation, or antigenic variation, was first described as it related to T1F and can be considered a method of host immune evasion, allowing bacteria to lower its expression of antigenic surface adhesions when switched off as well as to prevent coexpression and interference between surface components [75,76]. Here, the process is controlled by an invertible DNA element containing the promoter sequence for *fimA*. This sequence, which contains two inverted repeats flanking an intervening DNA segment, lines up with *fimA* to allow site specific recombination mediated by a recombinase enzyme and promotes transcription [77]. Expression through phase variation is thought to be regulated through a complex network of systems which responds to stress, and are likely to become activated during infection because of the hostile environment of the host urinary tract exerting pressure on invading UPEC [78]. The gene regulation which occurs during this response is regulated by a system of small-nucleotide alarmones, pGpp, ppGpp, and pppGpp (guanosine tetra- and pentaphosphates) and is produced by the enzymes RelA and SpoT [79,80]. Higher intrinsic levels of (p)ppGpp in *E. coli* result in high alarmone concentration, and this system activates the regulator *FimB* that controls the phase variation of type 1 fimbriae in *E. coli* [81].

#### 2.1.2. P Fimbriae

Like T1F, P fimbriae are extracellular protein appendages which play a role in the adherence of UPEC to uroepithelial cells [25]. They differ from type 1 fimbriae in that their adherence is mannose resistant, binding to globoseries glycosphingolipids as opposed to the mannose dependent adherence of type-1 fimbriae to mono-mannose structures [24]. They are also more associated with upper urinary tract infections and pyelonephritis (P fimbriae for pyelonephritis fimbriae) and are considered a particularly important virulence factor in UPEC, as they mediate attachment to epithelial cells in the kidneys and renal tubules [73,82]. P fimbriae are encoded chromosomally by the *pap* operon. The main fimbriae structure is encoded by *papA*, *papEF* encode adapter subunits and *papG* encodes the terminal adhesive protein [25]. Because of this, the *papG* gene is usually used as a molecular marker to determine the prevalence of P fimbriae among UPEC. Studies have reported the prevalence of the pap operon to be 14–28% in UPEC causing cystitis, 5–7% in commensal *E. coli* and 71–91% of UPEC isolated from patients with pyelonephritis [82,83]. Importantly, *papG* has four molecular variants, with distinct binding specificities. It follows therefore, that some of the molecular variants of *papG* create more virulent strains of UPEC for certain hosts, and several studies have sought to characterise the differences between these variants and the diseases they are associated with. For example, *PapGII* has been encountered more than other alleles in isolates from women with pyelonephritis, while PapGIII is found more commonly in UPEC which is causative of cystitis [84,85]. PapGI and PapGIV are encountered rarely and are less understood than the other two molecular confirmations. UPEC which are positive for the *papGII* gene has also been shown to be positive for additional virulence factors, which may contribute to its association with more severe, upper UTI [84].

Regulation of the *pap* operon by phase variation involves the formation of differential methylation patterns resulting from the competition between DNA adenine methylase (Dam) and leucine-responsive regulatory protein (Lrp) for binding to two sets of overlapping binding sites within the pap regulatory region [86]. The regulatory region is composed of two genes, the first is *papI* which activates the on-phase by interacting with Lrp-pap DNA complexes when a specific GATC sequences is methylated by Dam [87]. The second is *papB* which both activates *papI* transcription but also represses transcription when protein concentrations are high [88].

#### 2.1.3. S Fimbriae

S fimbriae are mannose-resistant adhesions which are encoded by the *sfa* operon. This operon consists of nine genes. The SfaA protein forms the dominant sub-unit, while SfaG, SfaH, and SfaS form minor sub-units, and SfaF forms the outer membrane translocation assembly protein [89,90]. Regulatory proteins SfaB and SfaC control expression, and like other fimbriae are manged by phase variation, that in this case, is influenced by temperature, osmolarity, glucose concentration, and other environmental factors [91]. S fimbriae has specificity for alpha-sialyl-2,3-alpha-galactose residues present on the glycoproteins of urothelial tissues in the bladder and kidneys [89,92]. Apart from urinary tract infections, S fimbriae-positive *E. coli* have been known to cause meningitis in new-borns, likely due to their binding potential for sialo-glycoproteins which are also found on brain micro vascular endothelial cells [93]. Studies which used molecular analysis to determine the prevalence of the *sfa* operon in *E. coli* have reported figures between 42–87% [60,67,68,94]. The *sfa* operon has been found more commonly in strains which form strong biofilms in vitro, as determined by tissue culture plate methods [94,95]. 

#### 2.1.4. The Afa/Dr Family of Fimbrial Surface Adhesins

The Afa/Dr family of adhesions are particularly associated with UPEC that cause cystitis in children, as well as pyelonephritis and recurrent urinary tract infections in young and pregnant women [96,97,98]. It is also regularly found in the genome of ST131, a particularly virulent UPEC clone [99]. Dr fimbria (diffuse adherence fimbria) are part of the Afa/Dr family of fimbrial surface adhesions and are separable from other adhesions by the wide range of binding specificity they possess. They can bind, firstly to the Dr blood group antigen on decay-accelerating factor (DAF), which is a glycoprotein present on the surface of uroepithelial cells [100]. Secondly, Dr fimbria can recognise carcinoembryonic antigen (CEA)-related cell adhesion molecules (CEACAMs) CEA, CEACAM1, CEACAM3, and CEACAM6 as binding receptors [101]. Interactions between Dr fimbria and these receptors have been shown to mediate internalisation and invasion of UPEC into the host epithelial cells [101,102]. 

The genes encoding afa/dr adhesions are composed of a primary transcriptional unit containing *afaA* which is a regulatory gene, *afaB* which encodes a chaperone protein, *afaC* which encodes an usher protein, *afaE* which encodes a major adhesion subunit, and a tip capping subunit protein encoded by *afaD*. This gene cluster is accompanied by a separate minor transcriptional gene; *afaF* [96]. Phase mediated expression of the afa/dr family adhesions is, like P fimbriae, mediated by differential methylation patterns caused by Dam and Lrp interactions in the afa/dr promoter region [103]. Activation, however, is carried out by integration host factor (IHF) and repression is mediated by H-NS, a histone-like nucleoid structuring protein [99]. Previously it has been shown that insertion sequences (IS) elements when inserted into the regulatory region of the *Afa/dr* operon cause increased expression in a ST131 UPEC clone [99].

#### 2.1.5. Non-Fimbrial Adhesins

Repeat-in-toxins (RTX) are a family of proteins usually transported to the cell surface through the type I secretory system [104]. They include toxins such as cytolysins and hemolysin, but also non-fimbrial adhesions [105]. Type one secretion A (*tosA*) is one such non fimbrial adhesion, encoded by the *tosRCBDAEF* operon [106]. Expressed on the cell surface, this adhesion can bind to human kidney and epithelial cells in the upper urinary tract [106]. The operon is regulated by TosR, with assistance from the global regulatory proteins H-NS and Lrp and its expression involves cross talk with the regulatory systems of other fimbrial adhesions, including Pap and Foc, allowing for cell surface organisation of both fimbrial and non-fimbrial adhesions [107]. Over expression of TosA has been shown to increase biofilm formation in vitro, in both LB broth and in human urine [107].

### 2.2. Curli

Curli is a functional amyloid protein that is an essential component of the biofilm matrix in many bacterial species, including *E. coli* [108]. Curli fibres are formed by the self-assembly of the major curli subunit protein, CsgA, which polymerises into fibrils on the bacterial cell surface upon contact with the minor subunit protein CsgB [109]. Curli production is regulated by the transcription factor CsgD, which activates the expression of the curli biosynthesis genes, which include *csgA*, *csgB*, and *csgG*, as well as genes involved in nucleotide biosynthesis and other metabolic pathways [65]. CsgE, CsgF, and CsgG proteins comprise the outer membrane curli secretion system, with CsgG forming a pore like outer membrane structure and CsgE and CsgF functioning as chaperones for secreted curli fibers [110]. 

The curli biosynthesis pathway in *E. coli* is particularly sensitive to changes in nucleotide biosynthesis, as disruption of the de novo purine biosynthetic pathway leads to a reduction in the intracellular level of cyclic-di-GMP, a secondary messenger molecule that is required for the activation of the *csgD* gene [111]. Cyclic-di-GMP is synthesised by diguanylate cyclases and degraded by phosphodiesterases, and its intracellular level is tightly regulated by a network of enzymes and signalling pathways [112]. The disruption of purine biosynthesis leads to a decrease in the intracellular level of cyclic-di-GMP, which in turn leads to a reduction in curli production and biofilm formation [64]. A study which created transposon mutants found that a strain with an insertion in the *purL* gene, resulted in a knockout variant which had reduced curli production and diminished biofilm formation [113]. The *purL* gene codes for formyl-glycinamide ribonucleotide amido-transferase (FGAR-AT), which is an enzyme that catalyses the fourth step of the purine biosynthetic pathway [114].

The involvement of the CsgD regulator in extracellular matrix production induced by extracellular nitrate suggests that the regulation of biofilm formation is also linked to the availability of nutrients in the bacterial environment. This was demonstrated by the creation of mutant strains lacking the membrane bound nitrate reductases NarGHJI and NarZYWV and produced increased biofilm as a result [115].

### 2.3. Biofilm Formation

Biofilm formation is a complex process that involves various genes and regulatory mechanisms. The fimbrial genes *fimB*, *fimE*, and the curli assembly component *csgE* are crucial for biofilm formation at all stages. However, during the initial 12 h of formation, genes related to gene transcription and motility play a more significant role along with these adhesion genes [116]. Several studies have investigated the relationship between phylogenetic groups and biofilm formation. Phylogroups B2 and D are most frequently associated with stronger biofilm formation or exhibit multidrug resistance and carry a high percentage of fimbrial, iron uptake, and toxin genes [117,118]. Moreover, one study reported no correlation between phylogenetic classification and biofilm formation, but a correlation was observed between antibiotic resistance and strong biofilm formation irrespective of phylogroup [81]. More generally, the biofilm formation of UPEC is variable between clonally distinct strains, and can include both weak and strong formers [119]. 

A study which characterised multidrug-resistant UPEC by phylogenic group found that adhesion genes were more commonly found in group B2 and C, and these strains had a higher resistance to fluoroquinolones, trimethoprim-sulfamethoxazole, and amoxicillin-clavulanate [30]. Among adhesin genes, the prevalence of *fimH* was the highest (91.8%), followed by *pap* (79.3%), *sfa* (12.0%), and *afa* (7.7%). Additionally, biofilm formation was significantly correlated with the *pap* adhesion gene [30].A study from Iran reported statistically higher levels of *papEF* in strong biofilm formers, and the adhesion genes *fimH*, *focG*, *sfaS*, *set-1*, and *cvaC* were more common in weaker biofilm formers [31]. We have previously shown that the biofilm forming tendencies of UPEC isolates are variable and not apparent from colony morphology, and that sub-inhibitory concentrations of ciprofloxacin and trimethoprim can, in some cases induce UPEC isolates to form stronger biofilms in vitro, even when resistant to antibiotics [119,120].

### 2.4. Toxin Production and Cytotoxic Effects

#### 2.4.1. Hemolysin

α-hemolysin (HlyA) is pore forming toxin and virulence factor produced by many strains of UPEC. The prevalence of hemolysin production by UPEC is between 21–47% of isolates, as determined by multiple studies [121,122,123,124]. The operon which encodes α-hemolysin consists of four genes, *hlyCABD* [125,126]. HlyA can trigger apoptosis in host cells by modulating cell death pathways and interfering with cell process regulators. For example it has been shown that protein kinase B (PBK) which inhibits apoptosis and stimulates immune responses in host cells, is itself inhibited by HlyA in human bladder epithelial cells [127]. The immune response is further disrupted because HlyA inhibits the metabolic enzyme ACLY, which results in low acetyl-CoA levels. This modulates histone acetylation and prevents the adequate promotion of proinflammatory cytokine and chemokine genes [128]. During infection, HlyA stimulates the upregulation of granulocyte-macrophage colony-stimulating factor (GM-CSF) which causes macrophage accumulation and kidney damage during episodes of acute pyelonephritis [34]. UPEC isolates that were found to be stronger biofilm formers in vitro have been shown to have higher hemolysin activity [122].

#### 2.4.2. Cytotoxic Necrotizing Factor Type 1 (CNF1)

Many strains of UPEC produce cytotoxic necrotizing factor type 1 (CNF1), an endotoxin that modulates the activity of host cells by interacting with the Rho family of GTPases and restricting their activation [129]. These GTPases act as molecular switches that control many cellular processes, such as the formation of actin structures, cell motility, cell cycle progression, and phagocytosis [130,131,132]. A range of effects on human and animal cell lines have been documented after exposure to CNF toxins, including elongation and multinucleation, which in vivo will lead to host cell damage and bacterial persistence [35]. There are four known variants of CNF: CNF1, CNF2, CNF3, and CNFY. They each work by deamidating Rho Q63, and the resulting inhibition of GTPase leads to a reorganisation of actin stress fibres, modification of the host cytoskeleton, and cell damage [133,134]. CNF2 differs from the chromosomally mediated CNF1 and CNF3 in that it is plasmid encoded [135].

CNF1 is encoded by the gene *cnf1*, which has been found in a pathogenicity island that also contains genes encoding P-fimbriae and α-hemolysin [136]. CNF1 internalises into host cells via receptor-mediated endocytosis and contains two distinct binding regions, one located on the N terminus composed of amino acids 135 to 164, and one on the C terminus composed of amino acids 683 to 730, the host receptor being laminin receptor precursor protein (LRP), which is expressed on the surface of eukaryotic cells [137,138]. 

Epidemiological data suggest that CNF1 is much more common in UPEC as compared to commensal *E. coli* isolates, and in mouse models it has been shown that CNF1 positive strains more extensively colonise the bladder, are recovered from the urine in greater numbers, and were better able to resist killing by human neutrophils than CNF1 negative strains [139,140]. CNF1 is also associated with *E. coli* strains that are causative of neonatal meningitis, as well as diarrhoea in children [141,142]. Regarding meningitis, it is thought that *E. coli* producing CNF1 can more effectively cross the blood–brain barrier and infect the brain, and has been shown to increase disease severity in a mouse model of meningitis [143,144]. 

#### 2.4.3. Serine Protease Autotransporter

SPATE (serine protease autotransporters of *Enterobacteriaceae)* are a subgroup of secreted proteins called autotransporters that contribute to the survivability and virulence of UPEC [145]. Among this family, the secreted autotransporter toxin encoded by the *sat* gene has particular importance to UPEC virulence due to its cytotoxic activity on urinary tract epithelial cells [146]. First discovered in *E*. *coli* CFT073, a strain isolated from a patient with acute pyelonephritis, it shows in vitro cytopathic activity in kidney and bladder cell lines and is found significantly more frequently in *E. coli* associated with pyelonephritis than in faecal *E. coli* strains [146]. When investigating the kidney damage caused by *sat* in a mouse model of UTI, Guyer et al. (2002) found that sat induces vacuolation in glomeruli and proximal tubules, a histological change that has been seen in other toxins such as VacA toxin of *Helicobacter pylori* [147,148]. *Sat* has also been shown to exhibit immunomodulatory effects, presenting proteolytic effects against complement proteins that would otherwise destroy invading bacteria once they enter the bloodstream following endothelial infection [36].

Table 2 shows virulence factors related to fimbrial and non-fimbrial adhesions with their corresponding genes and host cell receptors, as well as toxins produced by UPEC with associated genes and the corresponding effects on host cells.

### 2.5. Iron Acquisition Systems

Metal ions are essential nutrients required by UPEC for many crucial biological processes, including the formation of enzymes involved in cellular metabolism [150]. These are metalloenzymes, which require metals including iron, nickel, zinc and copper as co-factors, with iron being the most commonly required [151]. As iron is a key factor in the proliferation of UPEC within the urinary tract, and the retention of iron to deprive invading pathogens of the nutrient composes part of the defensive strategy on the part of the host, acquisition of iron is a key point of competition between host and pathogen [152,153,154]. UPEC have evolved several iron acquisition systems in response to this competition, the first being iron-chelating molecules called siderophores, of which there are four that UPEC can synthesis and secrete, those being enterobactin, salmochelin, yersiniabactin, and aerobactin [155]. Of these, enterobactin is the most conserved among clinical *E. coli* isolates, with the others appearing in various combinations [155,156]. These variations in genetic carriage of siderophore systems appear to be an important part of the UPEC immune evasion strategy, as host mechanisms can prevent iron acquisition activity of specific siderophores, for example, the innate immune protein lipocalin-2 (Lcn2) prevents enterobactin activity by binding ferric and aferric enterobactin [157]. Enterobactin is encoded by the operon *entABCDEF*; salmochelin is encoded by the genes *iroB* and *iroE*; yersinianactin is encoded by *ybtSETU*, *irp1*, and *irp2*; and aerobactin is encoded by *iucABCD*. The presence of *irp2* and *iuc* has previously been reported to be most prevalent in serogroups 025, 015 and 08 [52]. Rezatofighi et al. (2021) has reported that siderophores genes *fyuA* and *iutA* are found more frequently in group B2 than in other phylogroups [158] While iutD has been reported to be highly distributed into groups B2 and D among multidrug-resistant UPEC (MDR-UPEC) and extensively drug resistant UPEC (XDR-UPEC) [118]. In addition to siderophores, UPEC also utilise host haemoglobin to acquire iron using the outer membrane receptors ChuA and Hma [159,160]. Strains lacking either of these receptors has been shown to be ineffective colonisers of the kidneys in a mouse model of UTI, indicating their importance for successful colonisation of the upper urinary tract [160]. Even among asymptomatic bacteriuria strains, which lack many of the common virulence factors expressed by other UPEC, they will express iron acquisition systems, showing their necessity in urinary tract colonisation [161].

Fimbrial adhesions, curli, and non fimbrial surface adhesions are expressed on the cell surface to aid in colonisation and persistence through UPEC and host cell interactions, facilitating intra-cellular invasion as well as cell aggregation and biofilm formation. The expression of these surface proteins is regulated by phase variation. Outer membrane receptors and siderophores sequester iron which is essential for many cell processes and a scarce resource in the urinary tract. Secreted toxins cause tissue damage in an infected host and assist in immune evasion.

## 3. Antibiotic Resistance Mechanisms

**Figure 2:**
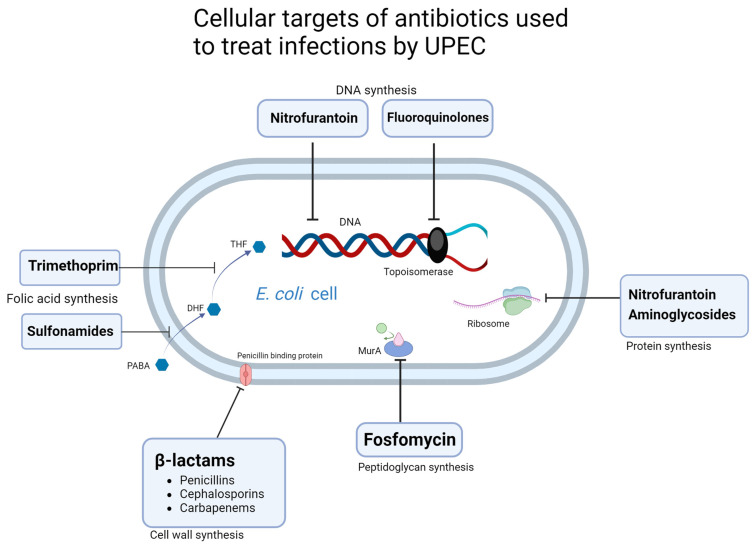
Cell targets of antibiotics used to treat infections by UPEC.

Antibiotic resistance mechanisms in UPEC can include chromosomal mutations in target genes and efflux pumps which allow for enhanced expulsion of antimicrobials from the cell interior. Additionally, enzymes can be produced which either deactivate the antimicrobial by degradation or by competing competitively for bindings sites. Here, the major mechanisms of resistance to clinically relevant antibiotics used in the treatment of UPEC-associated UTIs are described. These mechanisms are summarised in Table 3, and the cell targets of antimicrobials used in the treatment of UPEC associated UTIs are shown in Figure 2. 

### 3.1. β-Lactams

β-lactams are a wide category of antibiotics that include both wide and narrow spectrum penicillin, cephalosporins (first, second, third, fourth and fifth generation), monobactams, and carbapenems. Clinically, there are five classes: penams, cephems, carbapenems, clavams, and monobactams. They produce a bactericidal effect by binding to penicillin-binding proteins and inhibiting bacterial cell-wall formation [162]. Resistance to cephalosporins in UPEC can occur through various mechanisms, including β-lactamase production, target site mutations, and efflux pumps [163,164,165].

β-lactam resistance in UPEC is primarily due to the production of β-lactamases, enzymes that cleave the integral β-lactam ring and render these antibiotics ineffective. The most common type of β-lactamase produced by UPEC is CTX-M-type extended-spectrum β-lactamase (ESBL), which hydrolyses third generation cephalosporins and monobactams as well as other β-lactams [166]. A recent study by Mohammed et al. (2022) found that ESBL production was positively associated with certain serotypes, namely O8, O15, O21, O22, and O25 while being negatively associated with serogroups O1, O4, O7, and O16 [53]. Additionally, ampicillin resistance has been found to be significantly higher among serogroups O15 and O22 [10]. Molecular analysis of UPEC in Iran found that *bla^CTX-M^* and *bla^TEM^* genes were found in 53.2% and 45% of UPEC respectably, whereas the *bla^SHV^* gene was less commonly found at 5.4% [163]. These β-lactamase genes are often found on plasmids that can be easily transferred between bacterial strains that carry with them aminoglycoside, *dhfr* and *sul* genes. Additionally, a positive correlation has been observed between ESBL producing UPEC and the presence of biofilm formation genes *pgaA* and *pgaC* [167]. AmpC β-lactamase can confer resistance to aminopenicillins, cephalosporins, and monobactams, and they are not affected by clavulanic acid, an ESBL inhibitor [168]. In *E. coli*, AmpC production can be mediated by plasmids carrying *ampC* genes (pAmpC) [169], but also through chromosomal mutations. In this case, the promoter/attenuator region of the chromosomal cephalosporinase gene (*ampC*) is mutated, resulting in overexpression [170]. Carbapenems are antibiotics of last resort and the emergence of carbapenem-resistant UPEC (CR-UPEC) is an internationally recognised societal challenge [171]. The most common carbapenemase found in UPEC is the New Delhi metallo-β-lactamase (NDM), encoded by the *bla^NDM^* gene, which has been found in globally distributed UPEC isolates [172].

In addition to β-lactamases, UPEC can also acquire resistance through mutations in genes involved in cell wall synthesis. For example, mutations in the penicillin-binding proteins (PBPs) can lead to a decrease in the affinity of β-lactams for their targets, thereby decreasing their efficacy. Studies have characterised mutations in *mrdA*, the gene encoding penicillin binding protein 2, finding ten different patterns which increased in MIC of carbapenems [164] Mutations in *mrdA* have also been implicated in reduced activity of diazabicyclooctane, a B-lactamase inhibitor [173]. Efflux pumps are another mechanism by which UPEC can develop resistance to cephalosporins. The efflux pumps AcrAB-TolC and AcrAD-TolC have been shown to be over expressed in carbapenem resistant *E. coli*. One study found a strong relationship between AcrA over expression and ertapenem resistance, and AcrB over expression due to imipenem induced stress in *E. coli* [165]

Recent years have seen the introduction of novel β-lactam/β-lactamase inhibitor combinations for use against Gram-negative bacteria. Namely, ceftolozane/tazobactam and ceftazidime/avibactam. Both drugs combine a 2nd generation cephalosporin with an inhibitor which targets the active site of serine β-lactamases [174]. Both inhibitors, tazobactam and avibactam, effectively inhibit the activity of TEM-1, TEM-2, TEM-3, SHV-1, SHV-2, and CTX-M-14, However, neither will inhibit class B metallo-β-lactamases, such as NDM-1, and only avibactam shows effective inhibition of AmpC β-lactamase and *Klebsiella pneumoniae* carbapenemase (KPC) [174,175] This gives both antibiotic-inhibitor combinations a wide spectrum of activity against ESBL-producing UPEC, and meta-analysis of randomised control trials have indicated that the use of these drugs results in better outcomes for the treatment of complicated UTI when compared to alternative antibiotic options [176]. The main mechanisms of resistance for both drugs in UPEC are class B metallo-β-lactamases; however, other mechanisms that are not enzymatically mediated can still impart resistance, for example, mutations in PBP3, the target site for ceftazidime has been shown to render *E. coli* resistant to ceftazidime-avibactam [177]. Likewise, the efflux pump AcrAB has been demonstrated to increase the MIC of ceftolozane/tazobactam to *Enterobacteriaceae* previously [178].

### 3.2. Nitrofurantoin

Nitrofurantoin remains an effective first-line treatment and prophylaxis for lower UTIs due to the low resistance rates observed [179]. The slower development of resistance to nitrofurantoin compared with other antimicrobials may be due to the unique and incompletely understood mode of action. Bacteria first catalyse the breakdown of nitrofurantoin to several bioactive metabolites that possess antimicrobial properties using the bacterial oxygen-insensitive nitroreductases enzymes, expressed by the *nfsA* and *nfsB* genes [179,180]. The metabolites achieve their bacteriostatic affect by binding to bacterial ribosomes and inhibiting protein synthesis, as well as by inhibiting enzymes involved in the citric acid cycle and those involved in the synthesis of DNA and RNA [181]. The main mechanism of resistance to nitrofurantoin are deletion and insertion mutations in *nfsA* and *nfsB* genes. Additionally, resistance can be conferred due to mutations in the *ribE* gene which encodes for lumazine synthase, an enzyme which has involvement in the synthesis of the NfsA and NfsB co-factor flavin mononucleotide [182,183].

Resistance to nitrofurantoin can also be plasmid mediated. Firstly, the multidrug-efflux gene *oqxAB* is carried by a variety of plasmids types, and is commonly flanked by IS26 comprising the transposon Tn6010 [184]. While it remains unclear whether or not high-level resistance can develop from carrying the *oqxAB* gene alone, high level nitrofurantoin resistance results from *nfsA* inactivation combined with expression of the *oqxAB* gene [185]. Nitrofurantoin-resistant strains in fact show a higher prevalence of efflux genes than other resistant phenotypes, and 51% of them have been shown to be extensively drug resistant (XDR), exhibiting co-resistance to β-lactams, cephalosporins, carbapenems, aminoglycosides, and tetracyclines [186].

Recently, in a Welsh hospital, a second plasmid-mediated resistance mechanism was discovered—a mutated form of CTX-M-14 β-lactamase enzyme isolated from five UTI-positive urines. Three specific associated mutations (T55A, A273P, and R277C) were found to both significantly increase the minimum inhibitory concentration (MIC) to nitrofurantoin when recombinantly expressed in *E. coli* (Bio-85025) and to be capable of hydrolysing nitrofurantoin in a cell-free assay [187]. Potential dissemination of this gene, via horizontal gene transfer or clonal expansion, is an emerging clinical consideration.

### 3.3. Fosfomycin

Like nitrofurantoin, the resistance rates to fosfomycin among UPEC remains low, preserving its clinical utility in the treatment of UTIs [179,188]. The mechanism of action is also unique, meaning that the development of co-resistance is unlikely [189]. That said, resistance is increasing, especially among extended-spectrum β-lactamase (ESBL) producers [190]. Fosfomycin acts as a competitive inhibitor of the enzyme UDP-N-acetylglucosamine-enolpyruvyltransferase (MurA), which catalyses peptidoglycan synthesis in *E. coli*, by binding to the cysteine 115 residue [191]. Because of this, a C115D MurA mutant leads to ineffective inhibition and associated fosfomycin resistance [192].

For inhibition to occur, fosfomycin requires intracellular transport facilitated by the glycerol-30-phosphate (G-3-P) transport system (*glpT*) as well as the hexose phosphate uptake transport system *(uhpT)* [193]. Mutations in these transport genes have been shown to increase the MIC of *E. coli* for fosfomycin, but not to the degree where it is outside EUCAST susceptibility range, indicating that a multifactorial series of mutations and deletions in combination with other resistance mechanisms are required for fosfomycin resistance to develop through chromosomal gene mutations alone [194].

A series of plasmid-encoded metalloenzymes, *fos* genes, confer resistance by modifying fosfomycin to an inactive form, with the most common found in *E. coli* being *fosA3* [191]. A study in Mexico which determined the mechanism of resistance to fosfomycin among 350 ESBL-producing *E. coli* found that among the fosfomycin-resistant strains, 60.5% of them were *fos*-producing, with the majority of those strains carrying *fosA3*, while resistance due to mutations in the antibiotic transport system was responsible for 28.9% of resistant strains [195]. Recently, *fosA8* coding a protein with 80% identity to *fosA*, was identified from an *E. coli* isolated from urine, located on a transferrable plasmid and conferring high-level resistance [196]. This gene was traced to the genome of *Leclercia decarboxylata*, a species with natural fosfomycin resistance and a reservoir of transferable resistance genes [196]. Another plasmid mediated resistance gene, named *fosL1*, was identified from isolates in Switzerland and believed to be acquired by Tn7-related transposition. This gene shared 57% to 63% amino acid identity with other *fosA* genes [197].

Fosfomycin is available for use in both oral and intravenous formulations. Oral fosfomycin can be administered in the form of fosfomycin trometamol and fosfomycin calcium, while intravenous fosfomycin is formulated with disodium in a ratio of 1 g to 8 g with succinic acid as an excipient [198]. For oral formulations, the maximum concentration is reached in the urine after 2 h and will maintain a mean concentration above 128 mg/L for 36 h after administration with a 3 g dose [199]. While oral formulations are recommended as a first-line treatment for uncomplicated UTIs in women with infections caused by *E. coli*, is has also been shown to be effective in treating male UTIs and acute bacterial prostatitis [7,200]. This is due to the fact that, apart from the urine, elevated intraprostatic concentrations are also reached following administration [201]. 

Intravenous doses of fosfomycin, on the other hand, have a faster period of absorption and the urinary concentration will drop below 128 mg/L 12 h post administration [199]. The maximum plasma concentrations reached through IV administration are much higher, however, it has been shown that significant concentrations are reached at various body sites [199,202]. The favourable PK/PD profile coupled with the unique mode of action of fosfomycin, means that IV fosfomycin is particularly useful as a combination therapy with other antimicrobials. For example, it has been shown to be effective when used in combination with cefiderocol, a siderophore antibiotic resistant to several β-lactamases, including metallo-β-lactamases, to treat MDR Gram-negative bacteraemia in patients with impaired renal function [203].

### 3.4. Trimethoprim-Sulfamethoxazole

Trimethoprim inhibits the formation of tetrahydrofolic acid, a cofactor required for the successful synthesis of bacterial DNA. It is commonly paired with the sulphonamide drug sulfamethoxazole, which inhibits the synthesis of dihydrofolic acid [204]. Resistance to trimethoprim and sulfamethoxazole is highly associated with mobile genetic elements, plasmids, and integrons [205,206]. The genes which encode these resistance proteins have their ancestral roots in chromosomal *folA* and *folP*, from a species which have a natural resistance to trimethoprim and sulphonamides [207]. *Dfr* (di-hydrofolate reductase) are a large family of genes conferring resistance to trimethoprim. There are over 30 *dhfr* genes, with the most common in UPEC being *dfrA1* and *dfrA17* [208]. These *dfrA* genes are often found in close genomic proximity to *sul* genes—which confer resistance to sulfamethoxazole—within integrons and on plasmids. They are also commonly found among larger resistance islands that often contain *bla^CTX-M^*, chromate-resistance gene *chrA*, aminoglycoside genes *aac(3)-IIa*, *aac(6)-Ib-cr*, *aph(3′)-Ia*, and *aadA2* as well as other virulence genes *fimH*, *fyuA*, *irp2*, and *sitA* [209]. The inherent capacity for mobility associated with these resistance determinants has caused extensive spread of trimethoprim-sulfamethoxazole resistance among UPEC [205,210,211]. Selective pressure presented by previous antibiotic therapy has been shown to lead to the trimethoprim resistance, specifically the use of extended-spectrum penicillin, however, previous prescription with nitrofurantoin was found to be associated with a lower incidence of trimethoprim resistance [212].

Although these mobile determinants play the largest role in trimethoprim-sulfamethoxazole resistance, both efflux and chromosomal mutations have also been demonstrated to contribute to resistance to these agents. Regarding efflux, the AcrAB-TolC efflux system leads to increased trimethoprim MICs in *E. coli* and was shown to be upregulated due to the effects of decreased periplasmic glutathione concentration caused by loss-of-function mutations of the genes *gshA*, *grxA*, and *cydD*—known to regulate GSH content [213]. This leads to the speculation that mutations in these genes could mediate resistance to trimethoprim chromosomally. Furthermore, a study which screened a knockout library of *E. coli* found that *mgrB*, when deleted, resulted in trimethoprim resistance. *phoP*, *phoQ*, and *folA* were significantly upregulated in *mgrB*-deleted strains. This is because *mgrB* regulates the expression of *folA*, showing that many genes involved in folate metabolism can lead to trimethoprim resistance [214].

### 3.5. Fluoroquinolones

Ciprofloxacin, levofloxacin, norfloxacin, ofloxacin, and gatifloxacin are examples of fluoroquinolones used to treat UTI, and can have broad spectrum efficacy against both Gram-negative and Gram-positive bacteria [215]. The EAU does not recommend ciprofloxacin for uncomplicated UTI but recommends its use for cases of complicated UTI where the local resistance rates are below 10% and the patient has contraindications for third-generation cephalosporins or an aminoglycoside [7].

Fluroquinolones prevent bacterial DNA synthesis by inhibiting DNA gyrase and topoisomerase IV. DNA gyrase is responsible for introducing the negative supercoiling of double stranded DNA, while topoisomerase IV disentangles replicated DNA and allows for segregation of daughter chromosomes. DNA gyrase is composed of two subunits, GryA and GyrB, while topoisomerase is composed of four subunits, two ParC subunits and Two ParE subunits [216]. Chromosomal mutations to these targets, typically GryA, result in resistance to fluroquinolones. In a study of fluroquinolone-resistant *E. coli*, Esmaeel et al. (2020) found that 76.7% had mutations in the GyrA subunit, and the highest levels of resistance were associated with strains that possessed a dual Ser-83 and Asp-87 mutation in that subunit [217].

Resistance to fluoroquinolones is also highly dependent on the ability of bacteria to decrease drug accumulation by efflux, though different efflux systems vary in their contribution to resistance for different fluroquinolones. For instance, AcrB expression is significantly higher in ciprofloxacin-resistant *E. coli*, 26% higher than in susceptible isolates [218], although it is unlikely to be the predominant mode of fluoroquinolone resistance. Specific quinolone efflux pump genes *qepA* and *oqxAB* as well as plasmid-mediated quinolone resistance (PMQR) genes, which include *qnrA*, *qnrB*, *qnrC*, *qnrS*, *qnrD*, *qnrE*, and *qnrVC* are plasmid-encoded and can provide resistance in *E. coli* even in the absence of chromosomal mutations [219]. A study which looked at the distribution of *qnr* genes among ESBL-producing *E. coli* found that *qnrB* was present in 47.74%, *qnrS* was found in 47.10%, and *qnrA* was found in 2.58% of strains. These genes were highly associated with the ESBL genes *bla^CTX-M^*, *bla^TEM^*, *bla^SHV^* [220]. Another study found *qnrS* in 21 of 43 isolates, *qnrB* in 6 of 45 isolates and *qnrA* in 2 of 43 isolates [221]. They are highly transmissible and are transferred in tandem with additional β-lactams, gentamicin, and tetracycline resistance genes [222].

The most commonly reported PMQR mechanism is the AAC(6′)-Ib-cr enzyme which dually inactivates ciprofloxacin while additionally providing aminoglycoside resistance. It has been found to be present in >70% of strains from the subgroup sequence type ST-131, an antibiotic resistant clone that has been reported to be rapidly spreading and responsible for high rates of UTI and bloodstream infections [223]. While the enzyme alone imparts a low-level MIC increase for ciprofloxacin in *E. coli*, it enhances resistance when combined with resistance-associated chromosomal mutations [224].

DNA synthesis, protein synthesis, peptidoglycan synthesis, cell membrane synthesis, and folic acid synthesis can be inhibited by targeting key molecules using different antibiotic agents.

**Table 3:** Chromosomal, enzymatic, and efflux mediated resistance mechanisms commonly found in *E. coli* contributing to antibiotic resistance.

	Chromosomal Mutations	Enzymatic Mechanism (Plasmid Encoded)	Efflux Mediated
Nitrofurantoin	*nfsA*, *nfsB* [182] *ribE* [183]	CTX-M-14 (mutated) [187]	*oqxAB* [185]
Fosfomycin	*murA*, [192] *glpT*, *uhpT* [194]	*fosA*, *fosL* [195,196,197,225] (Various alleles)	
Trimethoprim	*mgrB* [214]	*dfrA1*, *dfrA5*, *dfrA7*, *dfrA12*, *dfrA17* [226] *dfrA8*, *dfrA14*, *dfr2d*, *dfrA3*, *dfrA9*, *dfrA10*, *dfrA24*, *dfrA26* [227]	*acrAB-tolC* [213]
Sulfamethoxazole		*sul1*, *sul2*, *sul3* [228]	
Fluoroquinolones	*gryA*, *gyrB*, *parC* [217]	*qnrA*, *qnrB*, *qnrC*, *qnrS*, *qnrD*, *qnrE*, *qnrVC* [219] *aac(6′)-Ib-cr* [223]	*acrB*, [218] *qepA*, *oqxAB* [219]
β-lactams	*mrdA* [164] *ampC* [92]	*bla^CTX-M^*, *bla^TEM^*, *bla^SHV^* [163], *bla^OXA^*, *bla^CMY^* [229] *pAmpC* [91]	*acrAB-tolC*, *acrAD-tolC* [165]

Table 3 shows resistant determinants of note for each antibiotic/antibiotic class stemming from either chromosomal gene mutation/deletion, antibiotic inactivation due to an enzymatic mechanism, or by efflux.

## 4. The Mobilome

### 4.1. Plasmids

Plasmids, which are extrachromosomal DNA molecules, can be classified and typed using various methods based on replicons [230], partition systems [231], *mob* genes for relaxases [232], and comparison of profiles generated through plasmid digestion with restriction enzymes [233]. Sub-typing can be used to further differentiate plasmids by double locus sequence typing [233]. The most commonly used typing method today is Inc/rep typing, which classifies plasmids based on their ability to stably coexist with other plasmids in the same bacterial strain—facilitated by the replication machinery within the plasmid [234].

Epidemic plasmids, such as IncF, IncI, IncA/C, and IncH, are the most abundant types of plasmids [235]. Among UPEC specifically, IncFIB, and IncB/O plasmid replicons are the most common [236]. However, the prevalence of certain plasmids differs depending on their sources and geographic locations [235]. These plasmid classes also appear to have different correlations with different antibiotic resistance genes, for example Incl1 plasmids are more often associated with clinically relevant strains and likely possess genes encoding ESBLs and AmpC enzymes [237]. More recently, the plasmid encoded colistin resistance gene *mcr-1* (phosphoethanolamine transferase) was detected on *E. coli* Incl1 plasmids recovered from Canada and Argentina [238]. IncF plasmids differ in that they can encode multiple replicons, and can carry *bla^CTX-M^*, *rmtB*, *oqxB* as well as *qnr* genes [239]. IncB/O plasmids, while less common, carry a great variety of resistance genes, including *bla^CTX-M−1^*, *bla^CMY−2^*, *bla^ACC−4^*, *bla^SCO−1^*, *bla^TEM−1^*, *sul1*, *sul2*, *aad*, *strA*, *strB* and *aacA4* [235].

Plasmids carry genes encoding resistance to nitrofurantoin [185] β-lactams [240], fluoroquinolones [222], aminoglycosides [241], trimethoprim [209], sulfamethoxazole [242], tetracyclines [243], and fosfomycin [195] among others. These genes are often distributed within plasmids in different combinations, allowing for the spread of multidrug resistance during horizontal transfer events [66].

A study which looked to determine the distribution of PMQR determinants among ciprofloxacin resistant UPEC isolates, as well as co-occurrence of ESBL production and β-lactamase inhibitor resistance, found that 50.0% of the isolates carried PMQR determinants, with ESBL production observed in 42.9% of these isolates and a β-lactamase inhibitor-resistant phenotype in 51.4%. Furthermore, co-occurrence and co-transmission of *aac(6′)-Ib-cr* with *bla^TEM^*, *bla^CTX-M^*, and *bla^OXA^* was the most prevalent, observed in 37.1% of the isolates [244]. An Egyptian study on the distribution of PMQR in UPEC found that the *aac(6′)-Ib-cr* gene was the most common (61.1%), followed by *qnrS* (43.3%) and *qnrB* (22.2%). The *qepA* gene had a low frequency (10%), while *qnrA* was not detected. The *aac(6′)-Ib-cr* gene was found to exist either alone or in combination with *qnrS* and/or *qnrB*. Interestingly, the coexistence of *qnrS* and *qnrB* genes was observed in only six isolates [217]. Another study characterised the plasmids of 25 UPEC each carrying one to two plasmids per strain and found that combinations of genes encoding β-lactamase, aminoglycoside modifying enzymes, dihydrofolate reductase, dihydropteroate synthase, and macrolide 2′ phosphotransferase were present in plasmids belonging to seven incompatibility groups, with IncFI and IncFII being the predominant resistance plasmids [66].

Studies have shown that plasmids can transfer between *E. coli* strains in a biofilm, and some plasmids have consistently higher transfer efficiency in biofilms when compared to liquid culture [32]. Another study focused on the effect of oxygen on IncP-1 plasmid transfer efficiency in biofilms, finding that the air-liquid interface can be a hot-spot for plasmid mediated gene transfer due to a high juxtaposition of donor and recipient cells [245]. The ability of plasmids to conjugate was also found to be dependent on biofilm biomass, with thinner biofilms allowing for increased frequency of conjugation. This suggests that the development of multidrug-resistant UPEC might be correlated with lower biofilm formation due to being a good recipient in conjugative transfer [246].

### 4.2. Integrons

Integrons are a mobile genetic element which assists in the horizontal transfer of multidrug-resistance genes in UPEC. Class 1 integrons can capture and express gene cassettes through a promoter (Pc) and primary recombination site. In addition, integrons can relocate within the host genome and other cells through their association with transposons and plasmids [247]. Integrons are common in *E. coli*, with one Chinese study reporting that 67.39% (93/138) of *E. coli* isolates carried class 1 integrons. Integrons have also been reported to be more common in UPEC than in commensal *E.coli* isolates [248].

The most common gene cassette found is *dfr* and *aadA*, conferring resistance to trimethoprim, streptomycin, and spectinomycin [247]. Other studies have indicated that there is a strong correlation between multi drug resistance and the presence of a class 1 integrons in UPEC [249,250,251]. A Mexican study which reported 63% of *E. coli* strains possessing the *ilt1* gene also found that the phylogenetic group B2 were more likely to have a class 1 integron. They went on to report the diversity of the cassettes finding eight variations with the *dfrA17* and *aadA5* arrangement present in 32.1% of variable regions [250]. Phylogenetic groups B2, A, and D have also been reported to harbour class 1 and 2 integrons in MDR-UPEC and XDR-UPEC, carrying the resistance genes *aadA1*, *aadB*, *aacC*, *ant1*, *dfrA1*, *dfrA17*, and *aadA4* [118]. Expression studies have shown a strong correlation between class 1 integrons and resistance to trimethoprim and cefotaxime [205].

The is also a strong correlation between *Int1* integrase and sulphonamide resistance, shown in one study were the authors examined 50 strains positive for the *IntI1*, and found that all of these strains were resistant to sulfamethoxazole, with the majority containing *sul1*, either alone or with *sul2* or *sul3* [252].

### 4.3. Transposons and Insertion Sequences 

Transposons and insertion sequences are further examples of mobile genetic elements that contribute to antimicrobial resistance in UPEC [228]. They do this by moving to new locations within a genome, or transferring from one genome to another, causing genetic rearrangements, interrupting genes, and carrying gene cassettes containing antibiotic resistance genes and virulence factors to plasmids, allowing for the horizontal spread of these genes to different bacterial species [253]. 

Insertion sequences are common in *E. coli*, with two or three per isolate and IS26 being the most common [254]. One study investigated the role of transposable elements in *E. coli* BH100, a strain closely related to UPEC strains, finding that IS4 and IS21 were associated with genomic islands containing genes associated with antimicrobial resistance and virulence. Additionally, the study found that a TN3 transposon harbouring β-lactamase TEM-1 was transmitted between plasmids of different replicon type [255]. Recombination events involving IS26 elements have been shown to impart the *qnr* resistance gene *qnrB19* to an *E. coli* plasmid isolated from animal origin [256].

The interruption of *nfsA* and *nfsB* genes was reported in UPEC studied in the United Kingdom, leading to nitrofurantoin resistance due to gene inactivation by the insertion sequences IS1 and IS10R, respectively [182]. Additionally, insertion sequences and transposons also play a role in the formation and evolution of cointegrate plasmids, which are plasmids formed by the fusion of two or more plasmids that share homologous sequences and can promote the dissemination of multidrug resistance. For example, IS1294 has previously been shown to mediate fusion between plasmids with different replicons, facilitating the co-transfer of *bla^CTX-M−55^* and *rmtB* [257].

## 5. Perspective Alternative Treatments for UTI

Developing and utilizing alternative therapies such as phage therapy, immunomodulation, vaccines, and novel antimicrobial agents offers a multifaceted approach to combat the challenge of escalating antibiotic resistance in UPEC. These alternatives can provide targeted, effective treatments for infections that are refractory to antibiotics, reduce the risk of further resistance development, and mitigate the adverse effects associated with antibiotic use. Presented here is an overview of potential novel treatments for UTI.

### 5.1. Vaccines and Immunomodulation Therapies

The development of vaccines for preventing UTIs is a dynamic and evolving field and recent studies have explored diverse strategies to harness the immune system’s ability to protect against UTIs. Previously, studies have investigated and found siderophores and heme iron receptors as promising candidates for antigens as part of a vaccine formulation [258,259]. In 2015, four antigens associated with iron acquisition (IreA, Hma, IutA, FyuA) were investigated and were found to generate a strong antigen-specific serum IgG which protected vaccinated mice, and demonstrated antibody binding to UPEC strains expressing those antigens [260]. Later in 2020, steps towards the optimisation of a vaccine using these antigens were made, and it was then was found that it provided the best protection against UPEC UTI when formulated with dmLT adjuvant [261]. Also, that year, J. Wu et al. investigated the use of intravesical vaccination with UPEC antigens and a Th1-skewing adjuvant to correct aberrant bladder immune responses. Intravesical immunization demonstrated marked protection against UTIs, attributed to its distinctive ability to recruit Th1 cells into the bladder. This approach proved effective even in mice with multiple UTIs and pronounced abnormal immune responses [262]. In 2022, a report from a multicentre randomised trial conducted by Lorenzo-Gómez et al. evaluated a sublingual bacterial preparation called MV140 for preventing recurrent UTIs in women. MV140 was found to reduce the incidence of positive urine cultures across bacterial species. Notably, the protection conferred by MV140 was durable for at least one year, setting it apart from short-term treatments, like antibiotic prophylaxis, that exhibit rapid loss of efficacy post-discontinuation [263]. Also in 2022, Kelly et al. introduced a supramolecular peptide nanofiber vaccine platform designed to elicit specific immune responses against UPEC. By assembling multiple pathogen-specific epitopes into nanofibers, the platform successfully generated robust anti-UPEC antibodies in mice. The resulting antibodies were effective against UPEC and demonstrated both systemic and mucosal presence. Furthermore, the nanofiber vaccine caused minimal disruption to the gut microbiota, a favourable aspect compared to antibiotics. Collectively, these recent studies showcase a range of innovative strategies in the development of UTI vaccines [264].

Aside from vaccines, there has been reported successes in using alternative immunomodulation therapies. These therapies aim to either stimulate or suppress specific components of the innate immune system to achieve a balanced and targeted response against bacterial infections [265]. These therapies target specific disease-related-immune-response regulators and have been demonstrated to be efficacious at controlling disease through modulating inflammation leading to more effective bacterial clearance from the kidneys and bladder in animal studies [265]. For example, inhibition of transcription factor interferon regulatory factor 7 (IRF-7) through the use of siRNAs was found to protected against infection and renal tissue damage in mice with pyelonephritis [266]. By the same token, the cytokine Interleukin-1 beta (IL-1β) has been found to be a key immune regulator in acute cystitis, and its suppression using IL-1RA anakinra as an inhibitor attenuated inflammation in infected mice, resulting in accelerated bacterial clearance [267]. 

### 5.2. Phage-Based Therapeutics

Phage-based therapeutics have several enticing advantages over antibiotics. For one, they have a comparatively lower potential for the development of resistance, owing to the fact that phages typically have a narrow host range, are strictly bactericidal, and mutational changes to phage receptors that cause resistance are also likely to impact fitness and virulence of bacteria [268,269]. Additionally, these mutations can lend itself towards increased sensitivity to antibiotics, for example, Phage U136B requires genes involved in the formation of LPS (*rfaC*, *rfaD*, *rfaE*, and *rfaP)* to successfully invade a host cell [270]. Mutations in these genes result in truncated LPS, and hyper sensitivity to several antibiotics [271]. Phages are also effective at controlling biofilms, especially when used in combination with other agents [272]. A particular utility for bacteriophages as an anti-biofilm coating for urinary catheters has been demonstrated previously [273]. For these reasons, there has been notable research interest in the development of a phage-based therapy for UTIs and there are many reported in vitro and animal studies on the use of bacteriophages in this regard [274,275,276,277]. Clinical studies, too, show promise. Recent reports include a case study reporting the successful treatment of recurrent ESBL *Klebsiella pneumoniae* urinary tract infection with the use of intravenous phage as a standalone therapy [278]. However, additional robust randomised clinical trials are required to properly assess the efficacy and safety of phage therapy and will be crucial to form a roadmap towards integrating phage therapy with traditional clinical practice [279].

### 5.3. Antipathogenic Agents

Antipathogenic agents can be used as part of an overall strategy to mitigate the potential to cause disease in UPEC, by interfering with certain virulence pathways, and in a sense, disarm virulent bacteria rather than interrupt their growth in the way antibiotics do. In this way, the selection for antibiotic resistance can be avoided [280]. For example, small molecule compounds have been developed to target type 1, P type, and S type adhesive fimbriae in UPEC [281]. These molecules are called pilicides, and can either directly prevent the assembly of the fimbria structure by binding to the associated chaperone proteins, or by inhibiting the transcription of fimbria genes [282]. Curlicides are small molecules which inhibit curli biogenesis, and include FN075, which was found to inhibit the assembly of type 1 as well as prevent the biogenesis of curli in UPEC, resulting in a less virulent and biofilm deficient strain following attenuation in a mouse model of UTI [283]. Quorum sensing is another virulent pathway which may be interrupted for therapeutic benefits, and a large number of compounds, including plant extracts, have been found which can reduce motility in bacteria, inhibit biofilm formation, or make biofilms more susceptible to antibiotics by affecting quorum sensing [284,285]. While these compounds have shown promising results to limit the pathogenic potential of UPEC in vitro, they are yet to be developed to the point where they can be formulated as a therapeutic. 

### 5.4. Nano Formulations

The application of nanotechnology to drug formulations can enhance the properties of conventional drugs, increasing their absorptivity, their specificity for target sites, and allowing for them to be attached to drug delivery systems [286,287]. Drugs formulated this way are typically 0 to 100 nm and possess unique features, such as a high surface to mass ratio, increased absorptivity, bioavailability, retention time, and reduced side effects over traditional macro formulations [286]. The benefit to antibiotic therapy is that, with nano-technology, the solubility and gastrointestinal contact area increases for oral formulations, and a higher uptake can be achieved through controlled and timed release of the bioactive ingredients [288,289]. Of particular interest, nano preparations could be useful in overcoming resistance mechanisms utilized by bacteria [290]. Antibiotics which are formulated smaller, or have appropriate materials incorporated with them can more easily transverse cellular membranes and access cellular components, and through massively increased drug accumulation within bacterial cells can overcome efflux mechanisms [291], [292]. They can also be formulated to exhibit multiple and varied modes of action, to make the development of resistance more unlikely as compared to the use of antibiotic in its molecular form [293]. Nanoparticle antimicrobials based on metallics (gold, silver, titanium, and zinc) have intrinsic antimicrobial properties and additionally are effective at eradicating biofilms, making them good potential candidates for the treatment of UTIs [294]. These metallic nanoparticles can also be used as drug carriers for existing antibiotics [291,295]. Delafloxacin-gold nano-formulation, a fluoroquinolone antibiotic using gold nanoparticles as a delivery vehicle was found to be more effective against Gram-negative and Gram-positive strains than delafloxacin alone, even when used at lower concentrations [295], and a ciprofloxacin-gold formulation has been found to overcome resistance mediated by efflux in multidrug-resistant *Pseudomonas aeruginosa* and *E. coli* [291]. That being said, the interactions between these metals and human tissues, as well as the effect of long-term exposure, requires further in vivo study.

## 6. Conclusions

UTIs caused by UPEC continue to have a significant impact on the global disease burden, and clinical treatment is considerably complicated by the development and spread of virulence factors and multidrug resistance. Resistance to most clinically useful antibiotics is often multifactorial, stemming from genomic mutations, inactivating enzymes harboured on extrachromosomal plasmids, and drug efflux systems. This is further aided by the genomic plasticity of the pathogen, which includes various mobile genetic elements such as plasmids, transposons, insertion sequences, and integrons. In order for clinical treatment to remain effective in the face of ever-increasing multi-drug resistance in UPEC, continued elucidation of the molecular factors which underpin resistance is necessary.

## Figures and Tables

**Table 1:** Treatment guidelines according to the European Association of Urology for Uncomplicated and Complicated UTI.

	Recommended First-Line Treatment	Recommended Empirical Treatments
Uncomplicated cystitis	Fosfomycin trometamol (Oral)	Trimethoprim
	Nitrofurantoin	Trimethoprim/sulfamethoxazole
	Cephalosporins (alternative)	
	Pivmecillinam	
Uncomplicated pyelonephritis	Fluoroquinolones	Ciprofloxacin
		Levofloxacin
		Trimethoprim/sulfamethoxazole
		Cefpodoxime or Ceftibuten
Complicated UTIs	Amoxicillin plus an aminoglycoside	3rd generation cephalosporin used intravenously
	2nd generation cephalosporin plus an aminoglycoside	

The guidelines in Table 1 are taken from the European urology association “Guidelines on urology infections 2023” [7]. First-line therapy refers to the initial recommended antibiotics for treatment of patients and are chosen with considerations given to antibiotic susceptibility results. Empirical treatment refers to antibiotics recommended during the period prior to the receipt of antibiotic susceptibility results. Uncomplicated UTIs are defined as acute, sporadic, or recurrent lower (uncomplicated cystitis) and/or upper (uncomplicated pyelonephritis) UTIs, limited to non-pregnant women with no known relevant anatomical and functional abnormalities within the urinary tract or comorbidities. Complicated UTIs include infections involving men, pregnant women, patients with relevant anatomical or functional abnormalities of the urinary tract, indwelling urinary catheters, renal diseases, and/or with other concomitant immunocompromising diseases.

**Table 2:** Adhesions and toxins in UPEC with associated genes and host interactions.

Adhesions	Genes	Host Receptor
Type 1 Fimbriae	*fimABCDEFGHI* [72]	D-mannose-containing receptors, uroplakins [23]
P Fimbriae	*papABCDEFG* [84]	mannose-resistant binding to globoseries glycosphingolipids [24]
Afa/Dr	*afaABCDEF* [96]	decay-accelerating factor, carcinoembryonic antigens [100,101]
S Fimbriae	*sfaABCDEFGHS* [149]	Sialo-glycoproteins [89,92]
Type one secretion A	*tosRCBDAEF* [106]	human kidney and epithelial cells [106]
Toxins	Genes	Effect in host
α-hemolysin	*hlyCABD* [125,126]	PBK and ACLY inhibition, GM-CSF upregulation [34,127,128]
Cytotoxic necrotizing factor	*cnf1* [136]	Inhibits Rho family of GTPases [129]
Serine protease autotransporter	*sat* [146]	Induces vacuolation in glomeruli and proximal tubules, degrades complement proteins [36,147]
